# Effects of *Shinbaro* pharmacopuncture in sciatic pain patients with lumbar disc herniation: study protocol for a randomized controlled trial

**DOI:** 10.1186/s13063-015-0993-6

**Published:** 2015-10-12

**Authors:** Jinho Lee, Joon-Shik Shin, Yoon Jae Lee, Me-riong Kim, Yong-jun Ahn, Ki Byung Park, Michael A. Kropf, Byung-Cheul Shin, Myeong Soo Lee, In-Hyuk Ha

**Affiliations:** Jaseng Spine and Joint Research Institute, Jaseng Medical Foundation, 858 Eonju-ro, Gangnam-gu, Seoul 135-896 Republic of Korea; Spine Center, Cedars-Sinai Medical Center, 444 S. San Vicente Blvd, Suites 800/901, Los Angeles, CA USA; LA Spine, Inc., 444 S. San Vicente Blvd, Suite 901, Los Angeles, CA USA; Busan National University, Yangsan campus, 49 Busandaehak-ro, Mulgeum-eup, Yangsan-si, Gyeongsangnam-do Republic of Korea; Division of Medical Research, Korea Institute of Oriental Medicine, 1672 Yuseongdae-ro, Yuseong-gu, Daejeon Republic of Korea

**Keywords:** Acupuncture, Pharmacopuncture, Usual care, Lumbar disc herniation, Protocol

## Abstract

**Background:**

Lumbar disc herniation is a major cause of sciatica and low back pain and imposes a heavy burden on both individual and society. While use of pharmacopuncture, a combined form of acupuncture and herbal medicine, for lumbar disc herniation is widespread in Korea and China, there is a paucity of research.

**Methods/Design:**

This study is the protocol for a three-armed, randomized, patient, physician, and assessor-blinded controlled pilot study. Sixty patients with severe non-acute sciatic pain diagnosed with lumbar disc herniation (NRS ≥ 5, onset between 4 weeks and 6 months) will be recruited and randomized 20 each to the *Shinbaro* pharmacopuncture (pharmacopuncture with acupuncture), acupuncture, and usual care groups, respectively. The 2 acupuncture groups will receive 2 sessions/week of acupuncture alone or with pharmacopuncture for 4 weeks (total 8 sessions), and the usual care group will receive conventional medication 2–3 times/day and physical therapy 2 sessions/week over 4 weeks (total 8 sessions). The initial acupuncture physician will administer acupuncture at 5 acupoints (GB30, BL40, BL25, BL23, GB34) in the 2 acupuncture groups, and mark an additional acupoint. A second acupuncture physician will administer pharmacopuncture to the marked acupoint in the pharmacopuncture group, and acupuncture in the acupuncture group during acupuncture needle retention. The second physician will administer acupuncture and pharmacopuncture in a similar manner in terms of advice and manual stimulation to maintain patient-blinding, treat the patient out of view of the initial physician, remove the additional acupuncture needle immediately, and cover the area with adhesive bandage to maintain physician-blinding. The primary endpoint will be at 5 weeks post-randomization, and the primary outcome will be Visual Analog Scale (VAS) of sciatic pain. Secondary outcomes will be VAS of low back pain, Numeric Rating Scale (NRS) of low back pain and sciatic pain, ODI, SF-36, EQ-5D, and PGIC. Post-treatment evaluations will take place 5, 7, 9, and 12 weeks after randomization.

**Discussion:**

This trial will evaluate the comparative clinical effectiveness of pharmacopuncture for severe non-acute sciatic pain patients diagnosed with lumbar disc herniation with usual care of conventional medicine and that of Korean medicine (acupuncture), monitor its safety, and serve as basis for a large-scale, multicenter trial.

**Trial registration:**

ClinicalTrials.gov NCT02384928, registered 27 February 2015.

## Background

Sciatica occurs due to lumbar disc herniation (LDH) in approximately 90 % of cases, [1] and is characterized by ipsilateral radiating leg pain [2] secondary to an inflammatory response from nerve root irritation [3]. The prevalence of sciatica ranges from 1.6 % in the general population to 43 % in specific working populations [4]. The annual incidence of sciatica in Western countries is estimated at 5 cases per 1,000 adults [5]. Although the natural history of sciatica is known to be favorable in most patients, [6] a sizable percentage (up to 30 %) report pain persisting for 1 year or more [7, 8]. LDH incurs considerable medical expenses, and according to Benefits by Frequency of Disease data from the 2013 Korean National Health Insurance Statistical Yearbook, the number of patients with intervertebral disc disorders (Korean Standard Classification of Diseases (KCD): M50-M51) was 2.5 million, and yearly medical expenses have reached 790 billion Korean won (excluding the Korean medicine sector of the KCD). Low back pain (LBP) was the most frequent disease in both outpatient and inpatient care in National Health Insurance reimbursements for Korean medicine, and the number of patients with intervertebral disc disorders (KCD: M51) was approximately 19,000 and reported medical expenses were 47 billion Korean won in 2013 [9]. As these statistics only include items eligible for reimbursement, total expenses are expected to be much higher taking into account non-reimbursable and indirect expenditures, and lost productivity.

Many randomized controlled trials (RCTs) comparing non-surgical treatment with surgery in LDH patients with sciatica have reported that between-group differences were not significant at long-term follow-up [10, 11]. Though guidelines and experts agree that first-line treatment for sciatica should be conservative, they are divided regarding specific treatment modality [7]. Recently, more multidimensional approaches are being considered in conservative treatment, and are not limited to conventional treatment, but include complementary and alternative medicine (CAM) [12]. According to a 2004 survey, 43 % of peripheral neuropathy patients use CAM to manage symptoms, and many patients cited dissatisfactory pain control with standard medical treatment as the reason for selecting CAM. Most commonly used CAM treatments included acupuncture, chiropractic manipulation, herbal medicine, magnetic therapies, and vitamins [13].

Pharmacopuncture is a treatment that combines two of the most frequently used Korean medicine treatment methods – traditional acupuncture and herbal medicine – by injecting herbal medicine extracts into acupoints. This method prolongs the mechanical and chemical effect of acupuncture and herbal medicine by optimizing acupoint access [14, 15]. Current usage of pharmacopuncture for musculoskeletal diseases in Korea may be roughly estimated from medical service use of patients with spine surgery sequelae at 2 spine-specialty Korean medicine hospitals where 69 % of 702 patients received pharmacopuncture. Taking into consideration the fact that 88 % received acupuncture, a reimbursable item [16], this data suggests that pharmacopuncture is used almost as frequently as acupuncture. Also, the “Korean Medicine Clinical Practice Guideline for Lumbar Herniated Intervertebral Disc in Adults” published by the Korea Institute of Oriental Medicine (a subsidiary organization of the Korea Research Council of Fundamental Science and Technology under the Korean Ministry of Science, ICT and Future Planning) includes pharmacopuncture in major treatment guidelines for Korean medicine doctors (KMDs), recommending bee venom and *Shinbaro* pharmacopuncture amongst others for LDH patients [17].

However, there is a paucity of rigorous RCTs on pharmacopuncture unbefitting its widespread use and inclusion in clinical guidelines for musculoskeletal diseases in Korea. Hence, we will evaluate the effects of a combined treatment of *Shinbaro* pharmacopuncture with acupuncture in non-acute sciatica patients due to LDH through: 1) comparison with usual care in pain severity, functional scales, quality of life, and adverse events to assess its comparative effect and safety; 2) comparison with acupuncture to determine the specific effect of *Shinbaro* pharmacopuncture; 3) comparison of acupuncture and usual care to evaluate the comparative effect of acupuncture; and thus appraise the feasibility of a large-scale clinical trial.

## Methods/Design

### Design and recruitment

This trial is a randomized, patient, physician, and outcome assessor-blinded pilot study with three parallel arms. Participants will be recruited at Jaseng Hospital of Korean medicine, situated in Seoul, Korea, from May to December 2015. Sixty sciatic pain patients with LDH as confirmed by MRI will be recruited from outpatients visiting Jaseng Hospital of Korean medicine, which is a spine-specialty hospital designated as such by the Korean Ministry of Health and Welfare (900,000 outpatient cases per year). Advertisements will also be placed on site at the hospital, online through Internet advertising media, and on the hospital website for further patient recruitment.

### Trial phases

Informed written consent will be obtained from participants at their first visit before data collection. The trial will consist of three stages.

#### Screening

Potentially eligible patients will be screened using inclusion/exclusion criteria. Magnetic resonance imaging (MRI) scans will be conducted to check correlation with physical examinations and confirm LDH diagnosis. Only MRIs taken after the onset of the current episode of sciatic pain will be considered relevant. Additional MRIs will be conducted at the investigation site as needed in participants with no MRIs for the current episode, or in discrepancy between and/or change in current symptoms and prior MRI. After confirmation by physicians of consistency of sciatic pain symptoms and MRI findings, the patient will be randomly allocated to one of three groups – *Shinbaro* pharmacopuncture, acupuncture, or usual care group. The patient will receive 8 sessions of the assigned treatment for 4 weeks. If the patient is on pain medication before enrollment, the medication will be discontinued for at least 1 day before baseline evaluation, and later replaced with a substitute medicine.

#### Intervention (4 weeks)

The *Shinbaro* pharmacopuncture group will receive *Shinbaro* pharmacopuncture and acupuncture, the acupuncture group acupuncture alone, and the usual care group physical therapy and conventional medication, respectively. Each group will receive 8 interventional sessions of *Shinbaro* pharmacopuncture, acupuncture, and physical therapy, respectively, twice a week over 4 weeks, and the usual care group will take an adjunctive 2–3 doses/day of conventional medication. All groups will receive four educational program sessions supervised by physicians once a week. Participants will be required to receive a minimum of six sessions of assigned treatment.

#### Post-treatment (8 weeks)

The primary endpoint will be at 5 weeks post-randomization, which marks the end of the 8 treatment sessions. Additional follow-up evaluations will be conducted after the primary endpoint. At the post-treatment phase, patients will be allowed pain medication intake, as needed, regardless of type or dose, and patterns of use will be investigated at each follow-up evaluation. To encourage attendance of follow-up evaluations and reduce number of drop-outs, all participants will have the choice to be provided with acupuncture and *Shinbaro* pharmacopuncture at the end of the trial phase (12 weeks post-randomization).

### Participants

#### Inclusion criteria

1. Age 25 years or older, and 55 years or younger
2. Sciatica patients with average sciatic pain on the Numeric Rating Scale (NRS) of 5 or higher during the 3 preceding days
3. Onset of at least 4 weeks to 6 months previous for current sciatic pain episode
4. Patients whose sciatic symptoms correlate with MRI findings of LDH
5. Patients who have agreed to follow the trial protocol

#### Exclusion criteria

1. Patients who have received invasive treatments (e.g. nerve blocks, pharmacopuncture, acupuncture) within the past week
2. Non-spinal or soft tissue pathologies which may cause LBP or sciatic pain (e.g. spinal tumors, rheumatoid arthritis)
3. Pregnancy
4. History of spinal surgery, or spinal pathologies other than LDH (e.g. spinal dislocation, fracture)
5. Severe progressive neurologic symptoms (e.g. cauda equina syndrome, progressive muscle weakness)
6. Patients for whom acupuncture may be inappropriate or unsafe (e.g. hemorrhagic diseases, blood clotting disorders, intake of anti-coagulation medicine, severe diabetes with risk of infection, severe cardiovascular diseases, or other conditions deemed unsuitable)

### Randomization and allocation concealment

Random numbers will be generated by a statistician unaware of the design and purpose of this study through stratified block randomization in random blocks of 3 (1:1:1) using a computerized random number generator to allocate patients into 3 groups. SAS version 9.1.3 statistical package (SAS Institute, Cary, NC, USA) will be used. The random numbers will be stored in sealed envelopes until an investigator opens each envelope for random allocation. In situations where code break is warranted during the study, such as serious adverse events (SAEs), the principle investigator will contact the other researchers. Randomization will be performed only after the patient has been confirmed to be eligible, received LDH diagnosis through both clinical signs and radiologic evidence, and given written consent for participation. The patients will receive explanations about the two types of acupuncture (*Shinbaro* pharmacopuncture and acupuncture) which will be conducted by KMDs in accordance with recommendations for pharmacopuncture in the “Korean Medicine Clinical Practice Guideline for Lumbar Herniated Intervertebral Disc in Adults” [17]. They will also be informed of the contents of usual care, which will consist of individually tailored physical therapy and conventional medicine from commonly used treatments for LDH in accordance with Korean Health Insurance Review and Assessment Service data. The envelopes will be opened in view of the patient, and to prohibit reuse of envelopes, the patient name and date of opening will be recorded on each envelope and stored in a double-lock cabinet for the entire duration of the trial under the care of a custodian not involved in the study.

### Investigators and blinding

#### Screening researcher

Once the patient has been determined to be eligible and provided written consent, patient data and baseline information will be collected by the screening researcher. Envelopes containing randomization information will be delivered by the custodian to the screening researcher, and after random allocation, the patient will be escorted to the acupuncture or usual care physician.

#### Statistician

The statistician will generate the random allocation table, and keeping the original, will give a copy to the second acupuncture physician.

#### Initial acupuncture physician

The initial acupuncture physician will provide patients in the two acupuncture groups with the initial acupuncture and education program.

#### Secondary acupuncture physician

The secondary acupuncture physician will verify the allocated group while the patient lies prone during needle retention from initial acupuncture, and administer *Shinbaro* pharmacopuncture or secondary acupuncture accordingly. The second physician will administer acupuncture and pharmacopuncture in a similar manner in terms of advice and manual stimulation, and limit conversation to that absolutely necessary for the procedure to maintain patient-blinding, treat the patient out of view of the initial physician, remove the additional acupuncture needle immediately, and cover the area with adhesive bandage to maintain physician-blinding.

#### Usual care physician

The usual care physician will prescribe the patient with case-appropriate treatments from the predetermined list of physical therapy and conventional medicine. If the patient was taking pain medication before trial participation, the usual care physician will consider prescribing the same dose of the same ingredient(s), and patients in the usual care group may be prescribed additional conventional medicine.

#### Outcome assessor

The outcome assessor will assess all outcomes at all time points (except adverse event assessments) in a separate room, and will not discuss treatment group allocation with the patient.

### Blinding

The outcome assessor and statistician will be blinded to the three different treatment types, and the patient and initial acupuncture physician will be blinded to the two acupuncture groups. If possible, visiting times will be scheduled so as to avoid patient contact between groups. The secondary acupuncture physician will not record any information in patient charts for secondary acupuncture-blinding in the initial acupuncture physician, patient, outcome assessor, statistician, and study monitoring agent. All researchers will be impartial towards each patient, and will not try to become knowledgeable of patient allocation. The acupuncture, pharmacopuncture, usual care, and patient education program will be conducted according to the predetermined standard operating procedure (SOP). Blinding will be maintained for study duration, and all data will be collected and transferred from paper case report forms to electronic data processing at study completion. Data will be cross-checked for accuracy, and all data will be non-accessible to all researchers except the statistician. A blinding test will be conducted to assess successful blinding in the two acupuncture group participants and the initial acupuncture physician after the second treatment session.

### Interventions

#### Initial acupuncture

Acupuncture will be administered by KMDs who have received 1) 6 years of university education, and 2) 4 years of specialist training at a Ministry of Health and Welfare-certified institution, or 3) have at least 3 years’ clinical experience at a Ministry of Health and Welfare-designated spine-specialty Korean medicine hospital.

The KMDs will receive five training sessions before trial initiation for standardized acupuncture administration following detailed SOP instructions. The acupuncture physicians and physician in charge of patient management in the usual care group will record symptoms and any recent changes in the patient chart at each visit, and conduct the once weekly education programs. The education program will inform the patient of the favorable prognosis of LDH, and give instructions for everyday activities and self-management. The education program will consist of handbooks, with weekly reminders and encouragement from the physician. However, the physician will maintain a neutral stance in answering questions about the effect of each treatment modality. KMDs assigned with patient education will also receive five prior training sessions to ensure homogeneity in education content. The 2 acupuncture groups will undergo a total 8 sessions of acupuncture, 2 sessions/week for 4 weeks. Disposable sterile needles (40 mm × 0.25 mm; Dong-bang Acupuncture, SeongNam, Korea) will be used. The initial acupuncture physician will mark 1 *Hyeopcheok* (*Huatuo Jiaji*, EX B2) point most relevant to patient symptoms with reference to the MR image using an indelible surgical skin marker (Viscot, East Hanover, NJ, USA) with an “x,” and will not administer acupuncture to that point but use 5 acupoints for needling (GB30, BL40, BL25, BL23, GB34) as recommended in the “Korean Medicine Clinical Practice Guideline for Lumbar Herniated Intervertebral Disc in Adults” [17]. The 5 points will be disinfected with disposable 70 % isopropyl alcohol cotton swabs, and needled to about 1-cm depth assisted by an acupuncture guide tube with no manual stimulation such as twirling or lifting and thrusting. In selecting left or right side in the 5 acupoints, GB30, BL40, and GB34 will be needled on the side with sciatic symptoms, and BL25 and BL23 on the contralateral back region. The reason for contralateral needling is to avoid overlapping with the area marked for secondary acupuncture or *Shinbaro* pharmacopuncture. The secondary acupuncture physician will disinfect the marked area with a povidic saturated swabstick (Gumi Pharma, Gimpo, Korea) using gradually expanding circular strokes to about a 5-cm radius. Needle retention will be standardized at 20 minutes. The initial acupuncture physician will leave a piece of paper marked with the patient’s random allocation number on the bed for the secondary acupuncture physician. The patient will remain in the prone position during acupuncture.

#### Secondary acupuncture –- Shinbaro pharmacopuncture group

The secondary acupuncture physician will administer pharmacopuncture at the acupoint marked by the initial physician in the *Shinbaro* pharmacopuncture group with no contact with the disinfected area. The secondary acupuncture physician will prepare for treatment by loading a disposable Kovax-syringe (20 ml, 23G x 1″; Korea Vaccine, Ansan, Korea) with 6 cc of refrigerated *Shinbaro* pharmacopuncture solution (prepared at Jaseng herbal medicine dispensary, an extramural facility meeting Korean Good Manufacturing Practice (K-GMP) standards), and replacing the syringe needle with a smaller gauge disposable needle (26G x 1 1/2″; Jung Rim, Jincheon, Korea).

The secondary acupuncture physician will recheck the allocation number and determine whether the povidone has dried, and after accessing the tissue perpendicularly at the marked area to a depth of 4 cm with the 4 cm needle, will slowly inject 6 cc of the solution. The physician will immediately remove the inserted needle while applying pressure to the surrounding tissue to reduce bleeding, and then apply pressure with dry sterilized gauze for about 15 seconds. A povidic saturated swabstick will be used for disinfection with gradually increasing circular strokes to about a 5 cm radius, and prevent wound infection with a Superpore wound dressing adhesive (9 cm x 12 cm; Band Gold, Gwangmyeong, Korea) with the added effect of concealing the area from the physician who will remove the other needles.

#### Preparation of Shinbaro pharmacopuncture

*Shinbaro* pharmacopuncture solution will be prepared at Jaseng herbal medicine dispensary following KMD prescription of a herbal medicine compound containing GCSB-5 (Eucommia ulmoides cortex, Acanthopanax sessiliflorum cortex, Achyranthis bidentata radix, Saposhnikovia divaricata radix, Cibotium barometz rhizoma), whose main properties of anti-inflammation, cartilage protection, and nerve regeneration have been demonstrated in vivo and in vitro, and Paeonia albiflora radix alba, Ostericum koreanum radix, Angelica pubescens radix, and Scolopendra subspinipes corpus (*Paeonia albiflora* twice the proportion of that of other ingredients). The ingredients will be extracted using reflux extraction with 70 % *spiritus vinosus*, then purified progressively (80 %, 90 %) using alcohol immersion and freeze-dried. The freeze-dried powder will be mixed and dissolved in filtered normal saline (0.9 % NaCl), filtered at 0.2 μm for microorganism removal, packaged in vials sealed with rubber stoppers, and sterilized at 121 °C for 15 minutes. Only end-products that have passed quality control inspection, such as microbe cultivation and contamination tests, will be used.

The freeze-dried powder concentration to be used in pharmacopuncture will be set at 1.178 mg/ml, which is within the interval of optimal anti-inflammatory effects and non-apoptosis in in vitro tests, and which has been tested in vivo for single-dose toxicity in beagle dogs and single-week and 4-week multiple dose toxicity in rats at a Good Laboratory Practice institution.

#### Secondary acupuncture – acupuncture group

The secondary acupuncture physician will administer acupuncture at the acupoint marked by the initial physician in the acupuncture group, accessing the tissue perpendicularly at the marked area to a depth of 4 cm using a disposable sterile needle (40 mm × 0.4 mm; Dong-bang Acupuncture, SeongNam, Korea) without an acupuncture guide tube, and with no contact with the disinfected area. The physician will adopt a manner highly similar to *Shinbaro* pharmacopuncture in method, procedure and timing to maintain patient-blinding. The physician will also use needle twisting to evoke soreness similar to injection of solution, and inform the patient that solution injection may cause soreness. The physician will immediately remove the inserted needle while applying pressure to the surrounding tissue to reduce bleeding, and then apply pressure with dry sterilized gauze for about 15 seconds. A povidic saturated swabstick will be used for disinfection with gradually expanding circular strokes to about a 5 cm radius, and prevent wound infection with a Superpore wound dressing adhesive with the added effect of concealing the area from the physician who will remove the other needles.

Administration of secondary acupuncture (*Shinbaro* pharmacopuncture or acupuncture) will take place within the 20-minute initial acupuncture needle retention.

#### Usual care

The usual care physician will provide patients with conventional medicine and physical therapy referring to a list of most frequently used treatments in patients with a primary diagnosis of LDH (KCD: M51, M541) from Health Insurance Review and Assessment (HIRA) 2011 statistics. Frequency and contents of medicine and physical therapy use will be recorded.

#### Co-interventions

Provision of LDH treatment (surgery, nerve blocks, other acupuncture, physical therapy, etc.) other than the interventions listed above will not be allowed. However, a maximum of 4 g of acetaminophen may be provided as rescue medication to all participants, and use will be recorded.

### Outcomes

The detailed time points of each outcome assessment index are listed in Table 1.

**Table 1:** Time points of each assessment index

Week	Screening (baseline)	1		2		3		4		5	7	9	12
Visit	1	2	3	4	5	6	7	8	9	10	11	12	13
Informed consent	o												
Demographic characteristics, Medical history	o												
Inclusion/Exclusion criteria	o												
Blood test	o									o			o
MRI	o												o
Credibility and Expectancy Questionnaire	o												
Blinding test				o									
Randomization		o											
Treatment		o	o	o	o	o	o	o	o				
Education program		o		o		o		o		o	o	o	o
NRS	o	o	o	o	o	o	o	o	o	o	o	o	o
VAS		o		o		o		o		o	o	o	o
ODI		o		o		o		o		o	o	o	o
Physical examination		o		o		o		o		o	o	o	o
SF-36		o								o	o		o
EQ-5D		o								o	o		o
PGIC										o	o		o
Safety assessment		o	o	o	o	o	o	o	o	o	o	o	o
Use of medication		o	o	o	o	o	o	o	o	o	o	o	o

EQ-5D EuroQol-5 Dimension, MRI magnetic resonance imaging, ODI Oswestry Disability Index, NRS, Numeric Rating Scale, PGIC patient global impression of change, SF-36 Short Form Health Survey 36, VAS Visual Analog Scale

#### Primary outcome measurement

The primary outcome will be intensity of sciatic pain as measured by the Visual Analog Scale (VAS). VAS uses a 10-cm line labeled at each end with scale anchors [18]. In pain measurement, patients are asked to mark a point that represents their pain between the anchors of “no pain” and “worst pain possible” (labels vary by study). Scores are recorded in millimeters with a total range of 0–100 mm [19]. As pain intensity may differ at rest and during activity, to reduce error patients will be asked: “Please rate the pain intensity you feel during daily activities.”

#### Secondary outcome measurements

Secondary outcomes will include intensity of LBP measured with VAS, and will likewise try to reduce error with the following question: “Please rate the pain intensity you feel during daily activities.”

LBP and sciatic pain intensity will also be assessed with NRS. Although NRS is considered to be a subjective index, it is widely used for its simplicity. In pain measurement, patients are asked to rate their pain by selecting a number from 0 to 10 that best represents their current pain severity (0 is “no pain”, and 10, “worst pain possible”) [20, 21].

Functional impairment will be evaluated using the accredited Korean version of the Oswestry Disability Index (ODI) [22]. The ODI is a 10-item questionnaire developed to assess level of disability due to LBP [23]. Each item is graded into 6 levels, each representing a score of 0–5. Higher scores indicate greater limitation relating to LBP.

For a more comprehensive evaluation of improvement in pain and functional disability, patient global impression of change (PGIC) will be assessed [20, 24]. PGIC grades the level of subjective improvement into 7 levels (1, very much improved; 2, much improved; 3, slightly improved; 4, no change; 5, slightly worse; 6, much worse; and 7, very much worse).

Health-related quality of life (HRQoL) of LDH will be assessed using the Short Form Health Survey 36 (SF-36) and EuroQol-5 Dimension (EQ-5D). SF-36 consists of 36 items across 8 domains: physical functioning, role-physical, bodily pain, general health, vitality, social functioning, role-emotional, and mental health [25]. SF-36 is used to rate functional health and well-being in patients and healthy individuals. Han et al. verified the reliability and validity of the Korean version of SF-36 in measuring HRQoL in aged Korean patients [26]. Higher scores indicate better HRQoL. EQ-5D is a tool developed for HRQoL assessment, and is widely used in the health care sector. Scores range from −1, “health worse than death” to 1, “perfect health” [27]. EQ-5D has 5 dimensions covering current health status and functionality: mobility (M), self-care (SC), usual activities (UA), pain/discomfort (PD), and anxiety/depression (AD), to be rated out of 3 grades (1, no problem; 2, some/moderate problem; 3, extreme problem). We will use weighted values in calculating EQ-5D, applying a weighted model for Koreans [28]:

$$ \begin{array}{c}\hfill EQ-5D\  index=1-\left(0.050+0.096\times M2+0.418\times M3+0.046\times SC2+0.13\times SC3+0.051\times UA2\right.\hfill \\ {}\hfill \left.+0.028\times UA3+0.037\times PD2+0.151\times PD3+0.043\times AD2+0.158\times AD3+0.050\times N3\right)\hfill \end{array} $$

M2, SC2, UA2, PD2, and AD2 will be assigned values of 1 if a grade 2, and 0 if not. M3, SC3, UA3, PD3, and AD3 will be assigned values of 1 if a grade 3, and 0 if not, and N3 a value of 1 if any of the 5 EQ-5D items is a grade 3, and 0 if not. Also, if all 5 EQ-5D items are grade 1, the EQ-5D index will be 1.

Range of movement (ROM) and straight leg raise (SLR) tests will be conducted to assess improvement following treatment. ROM measurements are valid (*r* = 0.97) and reliable (*r* = 0.94), [29] but not highly responsive (effect size = 0.1–0.6) [30]. While SLR measurements are reliable (intraclass correlation coefficient = 0.95), [31] with a sensitivity of 0.8 (72–97 %) and specificity of 0.4 (11–66 %), [32] they are also not very responsive (effect size = 0.2) [30]. If ROM is uncheckable from pain, the angle will be recorded as 0°.

Expectations of treatment will be assessed with the Credibility and Expectancy Questionnaire which uses a 9-point Likert scale [33]. Participants will be asked the following question at first visit (1, “not at all”; 5, “somewhat”; and 9, “very much”): “To what extent do you expect pharmacopuncture, acupuncture, and usual care therapy to relieve your symptoms?”

### Sample size

There are no prior RCTs which have assessed the effect of *Shinbaro* pharmacopuncture in sciatic pain patients due to LDH. Therefore, the purpose of this pilot study is to calculate the sample size and assess the feasibility of a larger trial. A total of 60 participants with 20 patients per group will be recruited in light of the relatively short treatment and follow-up period. Factoring in a 20 % drop-out rate, we anticipate that this will be the minimum recommended number required for a pilot study [34].

### Statistical analysis

The intention-to-treat and per-protocol analysis will be performed in all participants who complete the clinical trial with no major breach from the study protocol, and intention-to-treat will be the primary analysis. Missing values will be resolved using the last observation carried forward. Sociodemographic characteristics and treatment expectancy of study participants will be assessed by group. Continuous data will be presented as means and standard deviations, or medians and quartiles. One-way analysis of variance will be used if normally distributed, and Kruskal-Wallis test if not, and post-hoc analysis will be performed if there is a significant between-group difference. Categorical data will be expressed as frequencies and percents using chi-square test or Fisher’s exact test.

Analysis of covariance will be used to evaluate the primary outcome variable, VAS of sciatic pain at 5 weeks after randomization. Secondary outcome variables – VAS of LBP, NRS of sciatic pain, NRS of LBP, ODI, EQ-5D, and SF-36 -– will be assessed likewise. Between-group differences in PGIC and blinding assessment will use chi-squared tests.

Also, within-group differences before and after treatment (baseline and follow-up evaluations) will be examined using a paired *t* test or Wilcoxon signed rank test, and difference in time-by-treatment interactions using repeated measures analysis of variance. As an additional approach to quantify the difference among groups over total follow-up time, areas under the curve between the time of randomization and week 12 will be calculated and compared with 1-way analysis of variance.

All analyses will be performed using SAS version 9.1.3 statistical package (SAS Institute, Cary, NC, USA) with level of significance set at *p* < 0.05.

### Adverse events

Physicians will record all unexpected or unintended reactions at every patient visit in the *Shinbaro* pharmacopuncture, acupuncture, and usual care groups. The potential adverse effects of *Shinbaro* pharmacopuncture will include those known of acupuncture while keeping potential unknown adverse effects in mind. Physicians will rate the causal relationship of each treatment with the adverse event on a 6-point scale (1, definitely related; 2, probably related; 3, possibly related; 4, probably not related; 5, definitely not related; and 6, unknown), and classify all adverse events with the Spilker classification, which has 3 grades (1, mild: no intervention needed and does not greatly impede normal activity (function) of patient; 2, moderate: significantly impedes normal activity (function) of patient, and may need intervention, with subsequent resolution; 3, severe: severe adverse event needing intensive intervention, leaving sequela).

### Data handling and monitoring

Investigators will enter collected data into case report forms. Out-of-range or unclear entries and missing data will be recorded in data query forms, and sent to the investigational site for resolution. Data will be analyzed by baseline demographic characteristics, treatment effect, and safety assessment. Regular data monitoring will be conducted for quality control. Investigators will convene regularly to discuss any issues that may arise during trial operation, such as SAEs, protocol revision, and any important points raised by the investigators or participants.

### Safety monitoring

Safety assessments will focus primarily on frequency of adverse events including all SAEs. Adverse event information will be presented as number and percentage of participants who have experienced adverse events, and categorized by affected body region. Other collected information (e.g. severity, causal relationship) will be entered in safety monitoring reports.

### Stopping rules

Due to lack of report on pharmacopuncture safety, researchers and clinicians convened for discussion and review of inhouse safety records of pharmacopuncture patients for the past 4 years and existing literature on acupuncture safety [35] to assess potential adverse events which may require the participant to be dropped out from the study. Symptoms requiring early withdrawal from the study included local infection, severe progressive neurological disorders, nerve irritation/injuries, continued bleeding in area administered with acupuncture, and pneumothorax. The trial will be stopped if the principle investigator judges that there is unacceptable risk of SAEs.

### Ethics

This study has received approval from the Institutional Review Boards of Jaseng Hospital of Korean medicine in Korea (reference number KNJSIRB2015-08), and all investigators will adhere to the Helsinki declaration and Korean Good Clinical Practice guidelines, and receive three educational sessions on the protocol and SOP.

## Discussion

The purpose of this study is to assess the efficacy and safety of *Shinbaro* pharmacopuncture, which is included in LDH clinical practice guidelines, in severe non-acute sciatic pain patients with LDH. We will evaluate the specific mechanical and chemical effect of pharmacopuncture minus the mechanical effect of acupuncture at acupoints through comparison of pharmacopuncture and acupuncture. This study is also a practical comparative study comparing acupuncture, pharmacopuncture, and usual care, which are the most frequently used treatments for LDH in Korea.

Though clinical guidelines on LDH issued by the Health Council of the Netherlands [36] and the North American Spine Society [37] support use of conservative treatment as first-line treatment in absence of cauda equina syndrome, the efficacy of many conservative treatments for LDH remains unclear [38]. For determination of the contents of the usual care group for LDH in our study, we referred to several clinical guidelines and systemic reviews, but found conflicting evidence on the efficacy of various conservative treatments. While US national guidelines recommend epidural steroid injections for short term (2–4 weeks) pain relief, a report from the Korean National Evidence-based Healthcare Collaborating Agency analyzing the results of systemic reviews and National Health Insurance reimbursement claims states that evidence to make a recommendation for use of epidural steroid injections for pain or functional recovery is weak [39]. Also, evidence for or against other pharmacological and non-pharmacological treatments including non-steroidal anti-inflammatory drugs (NSAIDS), muscle relaxants, antidepressants, physical therapy, traction, and exercise was mostly insufficient or against use. We therefore decided to apply interventions most frequently used in actual clinical practice in Korea in selecting usual care group treatment rather than follow evidence-based guidelines. Korea has a government-operated national health security system, based on a health insurance scheme that covers the entire population and all medical facilities. The system reimburses approved medical expenses of most diseases including LDH, and the HIRA service database contains information on medication and medical service use, and diagnoses. The HIRA database is, therefore, a useful source of epidemiologic data and allows for nationwide population studies of various neurologic and non-neurologic diseases [40]. We summarized most frequently used treatments in patients with a primary diagnosis of LDH (KCD: M51, M541) in the 2011 HIRA National Patients Sample for physician reference in the usual care group in an effort to establish a rational and legitimate method of usual care.

The effect of pharmacopuncture is believed to be produced through a combined effect of placebo, needle stimulation, and the mechanical and chemical effect of the solution. We reasoned that evaluation of the specific effect of pharmacopuncture against the non-specific effect of acupuncture (placebo effect and needle stimulation effect) at matching acupoints would validate pharmacopuncture use as distinct from acupuncture. As acupuncture points and needle-type hold relevance in determining effect, we will assign acupoint selection for *Shinbaro* pharmacopuncture and acupuncture to the blinded initial acupuncture physician and administer acupuncture perpendicularly at the acupoint with an acupuncture needle of the same shape and size as *Shinbaro* pharmacopuncture needles. To strengthen blinding and induce a homogeneous placebo effect in groups, the second physician will evoke a sensation similar to that of pharmacopuncture stimulation by twirling the acupuncture needle and refrain from exposing any factors that may inform the patient of the method of treatment (e.g. advice, precautionary measures, disposal of used products) in accordance with the protocol and prior education and training.

Limitations of our study include that recruiting at a mainly Korean medicine-based hospital may lead participants to have higher expectations for pharmacopuncture or acupuncture than conventional treatment due to the cultural and medical environment of Korea. We will attempt to assess the effects through Credibility and Expectancy Questionnaires, and will discuss the results in the results paper.

GCSB-5, which is the primary ingredient of *Shinbaro* pharmacopuncture, has a long history of clinical use for spinal disorders such as LDH in Asia, and recent interest in natural products has led to pharmacological studies on its effects, showing that GCSB-5 modulates acute and chronic inflammatory processes, [41] has neuroprotective, nerve regeneration promoting and motor functional recovery accelerating properties by reducing oxidative stress, [42] and articular cartilage protective effects against progression of osteoarthritis [43]. Based on clinical experience and experimental evidence, GCSB-5 was developed as a pharmaceutical following prescription drug development standards and approved as a natural product for treatment of osteoarthritis by the Korean Food and Drug Administration in January 2011, [44–47] consequently providing evidence for clinical use in the form of pharmacopuncture. Analyses of outpatient data at 12 branches of a spine-specialty network Korean medicine hospital from December 2010 to October 2014 revealed that 289,860 patients (77.6 %) received pharmacopuncture an average 8.2 ± 12.3 times out of 373,755 outpatients, and of these, 104,445 (36 %) received *Shinbaro* pharmacopuncture, ranking second after bee venom pharmacopuncture. A 2010 survey on clinicians with at least 5 years’ experience of pharmacopuncture reported that 38 % used it to treat musculoskeletal patients to a depth of 15 mm using 1 cc or less [48].

Most pharmacopuncture studies are currently being conducted in Korea and China, and the majority are on bee venom pharmacopuncture. One clinical trial conducted in China reported that in treatment of LDH patients of blood-stasis type using *Gegensu Zhusheye* (puerarin pharmacopuncture), electroacupuncture and blood-letting cupping, using *Hyeopcheok* (*Huatuo Jiaji*, EX B2) points showed better improvement than that of *Ashi* points in symptom and pain relief, interleukin-6 levels and hemodynamics, [49] but the study was designed to differentiate the effect of different acupoints in the treatment group and control, and thus not well-equipped to observe the overall efficacy of pharmacopuncture. An analysis of the publication types of pharmacopuncture-related papers published in the journal “*The Acupuncture*,” registered in the National Research Foundation of Korea, showed that 438 pharmacopuncture-related papers were published up to August 2006, which takes up about 34.6 % of the total 1,268 papers published in the journal. Of these papers, 355 were non-clinical, 70 clinical, and 14 literature reviews, showing a large proportion of non-clinical papers [50]. The “*Journal of Pharmacopuncture*,” also registered in the National Research Foundation of Korea, has published 196 papers (73.9 %) on pharmacopuncture out of 265 manuscripts published up to June 2006 since its foundation in 1997, and though the percentage of clinical studies was higher at 35 % (67 papers), clinical study papers on pharmacopuncture in both journals were generally limited to observational studies and case series. The most frequently studied type of pharmacopuncture was bee venom pharmacopuncture in both journals, [51] illustrating the need for more clinical studies on different types of pharmacopuncture.

*Hyeopcheok* points are situated at or in close proximity to the nerve roots, and stimulation of this area is comparable to nerve root blocking therapy commonly used in conventional treatment for LDH. It can be surmised that the reason why *Hyeopcheok* points, in addition to regular meridian acupuncture points, has a long history of use in spinal disorders was at least partly due to a need to stimulate surrounding deep tissue and the nerve root that was being irritated from protruded disc tissue. While the current study aims to assess whether using pharmacopuncture is more effective at *Hyeopcheok* points than regular acupuncture, we will consider further comparisons with nerve root blocking therapy using steroidal injections. Comparative effect studies on bee venom pharmacopuncture, which has been rigorously studied and is widely used in Asia, should also be given due consideration.

While regulations for post-marketing surveillance of medicine and medical supplies do not apply to pharmacopuncture products, agreements to the aim of regulatory standard enforcement in products not obligated to receive post-marketing surveillance in pharmaceutical affairs laws have been reached by the Korean Food and Drug Administration and Ministry of Health and Welfare. Despite the fact that over 200 types of herbal medicine injections are available from pharmaceutical companies in China [52] and the market is fast growing in Korea also, with pharmacopuncture recognized in private insurance sectors such as car insurance, rigorous pharmacopuncture trials utilizing appropriate controls (placebo or active), randomization and blinding are scarce. This trial will evaluate the comparative clinical effectiveness of deep pharmacopuncture for severe non-acute sciatic pain patients diagnosed with LDH with the usual care of conventional medicine and that of Korean medicine (acupuncture), and apply randomization and group allocation concealment, with physician, patient, and outcome assessor-blinding to effectively remove bias. This study will be the first well-designed rigorous RCT to assess the comparative effectiveness of pharmacopuncture in accordance with Consolidated Standards of Reporting Trials (CONSORT) and Standards for Reporting Interventions in Clinical Trials of Acupuncture (STRICTA) guidelines, monitor its safety, and serve as the basis of a large-scale, multicenter trial.

## Trial status

This study is expected to recruit patients from September to April,2015, and be completed in September 2016.

## Abbreviations

AD: anxiety/depression
CAM: complementary and alternative medicine
CONSORT: Consolidated Standards of Reporting Trials
EQ-5D: EuroQol-5 Dimension
HIRA: Health Insurance Review and Assessment
HRQoL: health-related quality of life
KCD: Korean Standard Classification of Diseases
K-GMP: Korean Good Manufacturing Practice
KMD: Korean medicine doctor
LBP: low back pain
LDH: lumbar disc herniation
M: mobility
MRI: magnetic resonance imaging
NSAIDS: non-steroidal anti-inflammatory drugs
NRS: Numeric Rating Scale
ODI: Oswestry Disability Index
PD: pain/discomfort
PGIC: patient global impression of change
RCT: randomized controlled trial
ROM: range of movement
SAE: serious adverse event
SC: self-care
SF-36: Short Form Health Survey 36
SLR: straight leg raise
SOP: standard operating procedure
STRICTA: Standards for Reporting Interventions in Clinical Trials of Acupuncture
UA: usual activities
VAS: Visual Analog Scale
